# Nature Ambience in a Lunch Restaurant Has the Potential to Evoke Positive Emotions, Reduce Stress, and Support Healthy Food Choices and Sustainable Behavior: A Field Experiment among Finnish Customers

**DOI:** 10.3390/foods11070964

**Published:** 2022-03-26

**Authors:** Saara Vanhatalo, Hilkka Liedes, Kyösti Pennanen

**Affiliations:** 1 VTT Technical Research Centre of Finland Ltd., 02150 Espoo, Finland; saara.vanhatalo@vtt.fi (S.V.); kyosti.pennanen@gmail.com (K.P.); 2 VTT Technical Research Centre of Finland Ltd., 90590 Oulu, Finland

**Keywords:** food environment, stress, emotions, food choice, environmental sustainability, field experiment

## Abstract

Laboratory experiments have indicated that exposure to restorative ambiences in food environments can lead to beneficial outcomes for consumers, but there is little evidence if this positive effect holds true in real-life consumption conditions. Therefore, the aim of this study was to analyze the effects of lunch restaurant ambience on customers’ emotional responses, stress recovery, food choices, and generation of plate waste. The expectation was that ambience inducing positive emotional responses would lead to alleviated stress, healthier food choices, and reduced plate waste. A field experiment with a baseline and two experimental ambiences (‘nature ambience’ to induce positive emotions and ‘fast food ambience’ to induce less positive emotions) including visual and auditory stimuli was conducted in a lunch restaurant for one week per ambience. Emotional responses, and objective and subjective stress were measured from a subgroup of participants (*n* = 32). Food choices and plate waste were measured for all customers (*n* = 1610–1805 depending on the study week). During ‘nature ambience’ week, customers more often chose vegetarian dishes and generated less plate waste. The results on emotional responses and stress recovery were partially in line with the expectations. The study provides real-life evidence that restaurant ambience modification could lead to beneficial consequences for customers.

## 1. Introduction

Modern lifestyles in terms of mental workload, rush, and imbalances between work and personal life threaten people’s psychological and physical welfare, predisposing them to stress, unhealthy food choices and unsustainable behaviors, posing significant adverse effects on individuals and societies in terms of public health and the environmental sustainability of the food system. Stress alone, defined as a condition in which the perceived demands are not met by the resources of an individual [1], predisposing individuals to anxiety, depression and unhealthy lifestyle [2], was responsible for financial costs of 26 billion euros in the EU-15 in 2014 [3], with 62% of workers experiencing stress at least once a week [4]. In a similar vein, unhealthy eating habits have led to the trebling of global obesity rates since the mid-1970s, making obesity and being overweight larger killers than being underweight for most of the world’s population [5]. On top of these, unsustainable food consumption behaviors are partially responsible for the increased global CO_2_ emissions and therefore contribute to climate change [6,7]. 

The development of food environments where consumers make food choices and consume food offers one potential tool to deal with stress and improve eating habits both from the health and sustainability perspectives. There is plenty of evidence that food environment and context have effects on various aspects of consumers’ food consumption, such as (un)healthy food choices [8,9], food preference [10], and food product acceptance [11]. One potential explanation for the food environment’s effect on consumers’ behavior lies in its capability to generate different emotional responses in consumers [12]. This follows the idea of appraisal theories, which postulate that a person’s evaluation of an object (e.g., food environment) will lead to an emotional reaction with subsequent psychological (e.g., increased or decreased stress) and behavioral consequences (e.g., increased or decreased healthy food choices, more or less environmentally sustainable behavior) [13]. In general, it has been suggested that a positive emotional reaction will lead to more beneficial behaviors from the individual’s perspective, such as higher satisfaction [14] and increased quality evaluations of healthy snacks [12], while negative appraisals might lead to unbeneficial outcomes such as higher preference for products containing sugar [15].

Emotions have been shown to influence stress recovery, healthy food choices and sustainable behaviors. Restorative physical environments such as nature have been found to contribute to stress recovery by reducing negative mood states and enhancing positive emotions [1]. Already short nature visits [16] and even exposure to nature views on TV have been observed to generate positive emotional reactions and reduce stress [17]. In line with this, positive emotional states have also been observed to trigger healthy food choices [18]. Emotional reactions caused by the environment have an effect on sustainable behaviors as well. For instance, it has been found that exposure to restorative nature videos inducing positive emotional responses in comparison to videos about built environments inducing negative affective reactions promotes sustainable behaviors [19], and positive emotions in general are connected to sustainable behaviors [20]. In the sustainable food consumption context, negative emotional states are found to be among the factors explaining, for example, food wasting behavior [21]. These observations imply that stress recovery, healthy food choices and sustainable food behaviors could be promoted through a food environment that induces positive emotional reactions in people.

A notable part of the working age population consumes daily meals in lunch restaurants, defined as restaurants that mainly serve lunch and provide other food services, such as breakfast or cafeteria services, during office hours and are located on-site or in close proximity of business centers or major business areas. In 2015, it was estimated that approximately 17% of EU workers ate their lunch in on-site catering environments [22], making them attractive venues for the development of food environments to generate positive emotional responses and promote stress recovery, healthier food choices and sustainable food behaviors. Indeed, the restaurant servicescape (the physical environment of a service organization where the service transaction occurs) has been shown to influence consumers’ emotions and satisfaction [23]. Although the scientific evidence on the influence of the food environment on stress and food behaviors in a real food consumption environment is sparse [8], it is proposed that behaviors could be influenced by illumination, colors, odors and temperature modifications [24]. From this perspective, recent studies done in restaurant dining contexts have shown that multisensory lunch conditions including nature themed images, sounds and odors indeed enhance positive emotions among the study participants, but no effect on food choices was found [25], and that auditory stimuli that was perceived as “healthy music” increased healthy food choices [26]. However, the above-mentioned studies were carried out in laboratory settings instead of field conditions. 

The aim of this study is to analyze the effects of lunch restaurant ambience on customers’ emotional states, stress recovery, food choices and sustainable food behavior in a real-life consumption context in Finland. In comparison to earlier studies, the current study intends to make a contribution by adopting the logic of appraisal theories in the development of two experimental ambiences (‘nature’ to extract positive emotions and ‘fast food’ to induce less positive emotions) and by applying them in real-life conditions instead of a laboratory setting. The main expectation of the study is that the ‘nature’ ambience aimed at inducing more positive emotional responses leads to alleviated stress, healthier food choices and less plate waste in comparison to those ambiences which induce less positive emotional reactions.

## 2. Materials and Methods

### 2.1. Study Design

**Figure 1:**
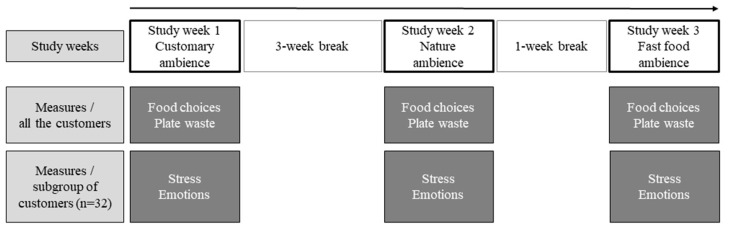
Overall study design with the study weeks, duration of breaks between the study weeks, measures for all customers, and measures for the subgroups of customers.

The study was conducted in TestEat, a research restaurant located in Helsinki, Finland. Although the restaurant is designed for research purposes, it is commercially operated by a large international food service provider and serves lunch for 300–400 customers every day from Monday to Friday throughout the year. The study ran during a seven-week period in spring 2019 with three actual study weeks (see overview of the study design in Figure 1).

There was a three-week break between study week 1 and study week 2 and a one-week break between study weeks 2 and 3. The reason for the unequal duration of the breaks was an attempt to avoid various holiday seasons (Finnish skiing holiday, Easter, 1st of May) during the study period, which might have had an effect on the restaurant menu, clientele amount and composition. The visual and auditory ambience of the restaurant was the customary one during the first study week, serving as the baseline measure, and it was modified during the following two study weeks (study week 2: ‘nature ambience’, with the aim to induce more positive emotional responses; study week 3: ‘fast food ambience’, with the aim to induce less positive emotional responses). The lunch menu, restaurant, and arrangement of the buffet line were kept constant during the study weeks. Photos of the arrangements in the buffet line and restaurant environment were taken every day in the first study week before the lunch service and were used every day in the coming study weeks to guarantee coherent arrangements between the weeks. No campaigns (price, marketing), which could influence customers’ food choices, took place during the study weeks or during the weeks between the study weeks. Food choices and the amount of plate waste of all the customers, as well as emotional responses to the restaurant ambience and the objective and subjective stress of a subgroup of customers (*n* = 32) were measured in all study weeks.

### 2.2. Participants

The sample for the study consisted of all the customers who purchased lunch in the restaurant during the study weeks (food choices and plate waste measurements) and a subgroup of customers (*n* = 32) who were recruited for the study and whose emotional responses to the eating situation, as well as objective and subjective stress, were measured throughout the study. 

#### 2.2.1. Subgroup of Customers Subjected to Emotion, Objective, and Subjective Stress Measures

Recruitment of the subgroup of customers was carried out in collaboration with the human resources department of a major employer located in the same building as the restaurant. Invitations to join the study were distributed through the company’s intranet, weekly newsletter and information screens located in the research restaurant and other locations in the office building. As the objective stress measures were based on heart rate variability, only healthy adults (exclusion criteria: chronic diseases influencing the functioning of the heart or disturbances of the mind potentially causing unknown variation in stress) were included in the study in order to control the stress measures. In addition, only those who ate lunch at least 3–4 days a week in the research restaurant were invited to participate in the study to ensure frequent exposure to the ambiences and to obtain as much relevant data as possible for the emotion and stress measures. Volunteers were guided to fill in an online screening form and those who met the inclusion criteria were invited to participate in the study. At the info visit, the volunteers were given a detailed written description of the study and the information was summarized verbally by the study personnel. After that, the participants signed informed consent forms, and received study IDs and activity bracelets (Garmin, Vivosmart 4) for objective stress measurement. Detailed written instructions about the use of the activity bracelets were provided. The participants were instructed to wear the bracelet in the non-dominant hand superior to the carpal bones and to attach the bracelet firmly, but not tightly, during the working hours in the study weeks. Before the study, the participants were instructed to start using the activity bracelet 3 days in advance to get comfortable with the use. One participant withdrew from the study due to workplace change. Thirty-two participants (21 females, 11 males, 45.6 ± 9.2 years) completed the study. After the study, the participants were given three movie tickets and a voucher (total worth 50 euros) to compensate for the time and effort used for the study. 

#### 2.2.2. All Customers Subjected to Food Choice and Plate Waste Measures

The other study population consisted of all the restaurant clientele during the study weeks. They were mainly office workers of the office building in which the restaurant is located, their visitors, as well as members of the general population living in the neighborhood. The sample sizes for each study week were calculated according to the transactions recorded in the restaurant sales data. In study week 1 (baseline week), 1610 lunch purchases were recorded, followed by study week 2 (‘nature ambience’ week) with 1714 purchases and study week 3 (‘fast food ambience’ week) with 1805 purchases. The customers were not informed about the ongoing study. However, the restaurant personnel were instructed to provide an information leaflet about the study with the researchers’ contact information for further queries to those customers who might ask about the changes in the restaurant during the study. At the end, no customers contacted the research group.

The ethical committee of VTT Technical Research Centre of Finland Ltd evaluated and approved the study design and experimental procedure (121739/Opportunity_FE_4.0_2019, approved 16.1.2019). The study was conducted according to the ethical principles of good research and clinical practice described in the declaration of Helsinki.

### 2.3. Ambience Design

The visual appearance, soundscape, and brightness of the general lighting in the restaurant were modified to create two unobtrusive and immersive experimental ambiences: ‘nature ambience’ to induce a positive emotional response and ‘fast food ambience’ to induce a less positive emotional response. The choice of the ambiences was based on previous research indicating that nature ambiences (including visual appearance and soundscape) are perceived restorative [16,17], induce positive emotional reactions in humans [12,27,28] and thus potentially contribute to stress recovery, healthy food choices and sustainable behaviors (i.e., plate waste in this study). On the contrary, exposure to colors with longer wavelengths (red, yellow, orange) in abstract forms is considered as arousing [29] and fast-paced instrumental music has been shown to lead to sympathetic arousal [30] and to delay recovery from stress [31]. In terms of lighting, it has been proposed that warm ambient light has a relaxing effect on humans while bright general lighting contributes to arousal and activation [24].

A pre-test was conducted to confirm that the images and soundtracks (images purchased from Adobe Stock library under standard license and sounds from the FMA Free Music Archive and from the YouTube Audio Library under the Creative Commons license) representing ‘nature ambience’ and ‘fast food ambience’ induced opposite emotional responses. Eighteen volunteers were recruited from the staff of VTT Technical Research Centre of Finland Ltd., Espoo, Finland. They were presented with four distinct nature images as image pairs constituting eight pairs of nature images and four red-yellow-orange abstract images as image pairs constituting eight pairs of abstract images in randomized order on paper. In addition, they were played two soundtracks with birds singing and three soundtracks with fast-paced instrumental music. The volunteers were asked to evaluate the calmness (positive emotion) and restlessness (negative emotion) evoked by the image pairs and soundtracks one after each other on a visual analogue scale (VAS) from 0 to 10, with higher values indicating calmer (or more restless) feeling.

**Figure 2:**
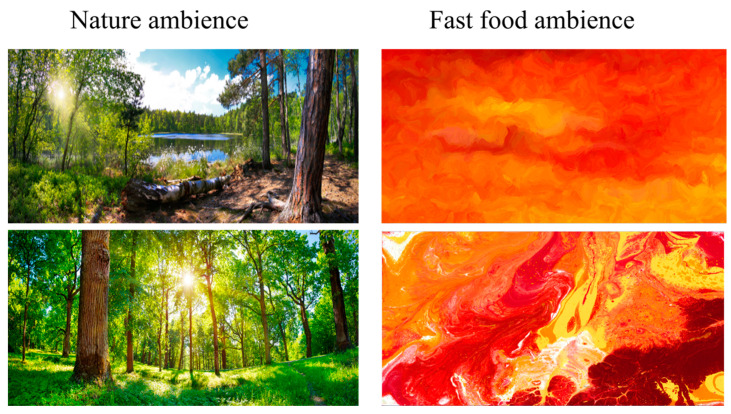
Image pairs chosen for the experimental ambiences.

The evaluations of calmness evoked by the nature or abstract image pairs ranged from 2.6 ± 2.2 to 8.9 ± 0.9 on a scale from 0 to 10 in which 0 = does not evoke calmness at all and 10 = evokes calmness a lot. Evaluations of restlessness ranged from 1.0 ± 1.1 to 6.9 ± 1.9 on a scale from 0 to 10 in which 0 = does not evokes restlessness at all and 10 = evokes restlessness a lot. The image pair evoking the least intensive restlessness (1.0 ± 1.1) and the most intensive calmness (8.9 ± 0.9) was chosen to represent ‘nature ambience’. The pair evoking the most intense restlessness (6.9 ± 1.8) and the least intensive calmness (2.6 ± 2.1) was chosen to represent ‘fast food ambience’ (Figure 2). There were statistically significant differences between the chosen ‘nature ambience’ and ‘fast food ambience’ image pairs regarding evoked calmness and restlessness (*p* < 0.001 for both). 

Birdsong soundtrack 1 scored 8.4 ± 1.9 in calmness on a scale from 0 to 10 in which 0 = does not evoke calmness at all and 10 = evokes calmness a lot and 1.5 ± 1.8 in restlessness. Birdsong soundtrack 2 received calmness evaluations of 3.4 ± 1.9 and restlessness evaluations of 5.9 ± 2.1 on a scale from 0 to 10 in which 0 = does not evokes restlessness at all and 10 = evokes restlessness a lot. Based on this result, birdsong soundtrack 1 was chosen to be used for ‘nature ambience’. Fast-paced music soundtrack 1 scored 4.3 ± 2.6 in calmness and 5.5 ± 3.0 in restlessness. Fast-paced music soundtrack 2 received a calmness evaluation of 4.5 ± 2.3 and a restlessness evaluation of 5.2 ± 2.5. Fast-paced music soundtrack 3 scored 5.2 ± 1.7 in calmness and 3.9 ± 2.5 in restlessness. The soundtracks did not differ regarding the evoked calmness. The soundtrack evoking the most intensive restlessness was chosen to be used in ‘fast food ambience’ (soundtrack 1). There were statistically significant differences between birdsong soundtrack 1 and fast-paced music soundtrack 1 regarding evoked calmness and restlessness (*p* < 0.001 for both). 

**Figure 3:**
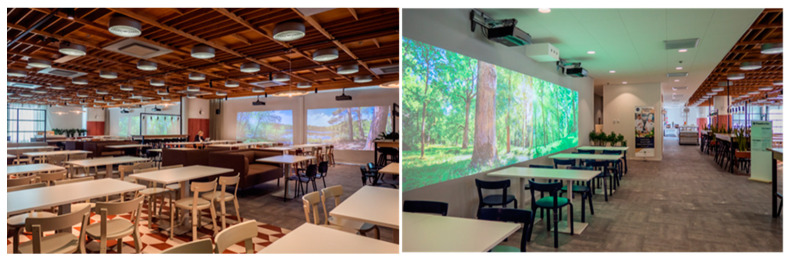
Illustration of ‘nature ambience’ projections in different parts of the lunch restaurant.

The practical application of ambiences in the lunch restaurant included three elements: (1) visual images projected on the walls (‘nature ambience’: nature images; ‘fast food ambience’: red-yellow-orange abstract images), (2) soundscape (‘nature ambience’: birdsong; ‘fast food ambience’: fast-paced instrumental music), and (3) general lighting (‘nature ambience’: slightly dimmed; ‘fast-food ambience’: full lightning). The visual ambiences in the restaurant were created by presenting four large still image pairs (approx. 6 m^2^ each) on the restaurant walls by using multiple ultra-high-definition projectors (Figure 3). The volume of the birdsong soundtrack in the ‘nature ambience’ week and the fast-paced instrumental music soundtrack during the ‘fast food ambience’ week was adjusted to 46–47 dB. During the ‘nature ambience’ week, general lighting was slightly dimmed (to retain constant lighting throughout the week, the correct setting was marked in the dimmer) while during ‘fast food ambience’ week, full lighting was on all the time [24]. The curtains were kept closed during all study weeks to exclude the possible influence of changing seasonal and weather conditions on customer behavior. 

### 2.4. Measures

#### 2.4.1. Emotions

The subgroup of participants (*n* = 32) was asked to evaluate their emotional states just after lunch through an online questionnaire administered in QuestBack software. The link to the emotion questionnaire was sent to the participants by email at 11 a.m. each study day. The intensities of the positive (contentment, joy, calmness) and negative (worry, discontentment) emotions were measured on a scale from 1–5 (1 = not at all, 2 = a little, 3 = somewhat, 4 = a lot, 5 = very much) adapted from the Consumption Emotions Set (CES) scale [32]. Based on the measures, composite variables for the analyses were formed and Cronbach’s alphas were calculated to check the reliability of the formed variables for positive emotions (‘customary ambience’ week α = 0.71, ‘nature ambience’ week α = 0.83, ‘fast food ambience’ week α = 0.91) and negative emotions (‘customary ambience’ week α = 0.77, ‘nature ambience’ week α = 0.66, ‘fast food ambience’ week α = 0.92).

#### 2.4.2. Objective and Subjective Stress

Objective and subjective stress were measured from the subgroup of participants (*n* = 32) recruited for the study. Heart rate variability (HRV) based data were collected through Garmin Vivosmart 4 activity bracelets as an objective indicator of stress [33]. The data were recorded for the entire working day, but for the analysis, data from 180 min before and 180 min after lunch were used. The activity bracelet measured stress level on a scale from 0 to 100 (0–25 = resting state, 26–50 = low stress, 51–75 = medium stress, 76–100 = high stress). The stress data provided by the bracelet were first synchronized with a Garmin Connect application in the participants’ mobile phones and then synchronized with Garmin servers. From there, the data was transferred through the Garmin Health API to study servers from where the research group extracted the data.

Subjective stress was measured with a questionnaire, which participants were instructed to fill in right before and after lunch. A link to the pre-lunch stress questionnaire was sent to the participants by email each study day at 10 a.m. The link to the post-lunch stress questionnaire was sent to the participants by email at 11 a.m. each study day. In the questionnaire, the participants were asked to evaluate their level of positive stress (excitement, motivation, vitality) and negative stress (anxiety, restlessness, nervousness) on a scale from 1 to 5 (1 = not at all, 2 = a little, 3 = somewhat, 4 = a lot, 5 = very much). Composite variables for analyses were developed and Cronbach’s alphas were calculated to check the reliability of the formed variables for positive stress before lunch (‘customary ambience’ week α = 0.79, ‘nature ambience’ week α = 0.77, and ‘fast food ambience’ week α = 0.86), positive stress after lunch (‘customary ambience’ week α = 0.84, ‘nature ambience’ week α = 0.90, ‘fast food ambience’ week α = 0.80), negative stress before lunch (‘customary ambience’ week α = 0.72, ‘nature ambience’ week α = 0.87, ‘fast food ambience’ week α = 0.72), and negative stress after lunch (‘customary ambience’ week α = 0.80, ‘nature ambience’ week α = 0.80, ‘fast food ambience’ week α = 0.79).

#### 2.4.3. Food Choices and Plate Waste

During the study, the lunch restaurant offered five different lunch options each day, including two vegetarian warm dish options and three warm meat-based dish options (fish, red meat or chicken being the main protein component in these dishes). In addition to the indicated main components, a typical dish also included salad from the buffet line, a carbohydrate component (e.g., rice, pasta, potato), bread, drinks (e.g., water, milk, juice), dessert, coffee, or tea. According to sales data, the number of purchased vegetarian dish options for each week were summed up to represent healthier and more sustainable meal choices [6]. The rest of the choices were combined to represent meat-based and less healthy and less sustainable meal choices and summed up for each week. 

For the plate waste measure, the restaurant personnel weighed the total amount of plate waste at the end of the lunch service in each study day. To increase the reliability of the weighing, the personnel were trained by the researchers before the data collection. In addition, they were provided written instructions and a template onto which the weighing results were marked every day. The average amount of plate waste per customer per day was calculated based on the total weighed plate waste divided by the number of customers in each day. 

### 2.5. Data Analysis

#### 2.5.1. Pre-Processing of the Stress Data

As the lunch time for the participants in the recruited subgroup varied, the time points for “before lunch” and “after lunch” were defined for each participant for each study day using the time stamp of their answers to the pre-lunch and post-lunch questionnaires. For the study, it was crucial that the questionnaire answers were given just before or right after the lunch to get an understanding of the momentary stress and emotional states and the potential effects of lunch ambience on those. Therefore, the time stamp data were pre-screened to check that the times were valid. The following principles were used to pre-screen the data: (1) If the pre-lunch questionnaire was filled in 15 min before or during the opening time of the lunch restaurant and the post-lunch questionnaire was filled in by a latest of 90 min after the opening time and the duration of lunch (15–90 min) seemed reasonable, the time stamps were used as such. (2) If there was only one time stamp available and the timing was reasonable (as earlier), the time stamp was used as such. (3) If the first time stamp was more than 15 min before the opening time of the restaurant, the time stamp was replaced with a time stamp where 1 h was subtracted from the post-lunch questionnaire time stamp. (4) If the interval between time stamps was less than 15 min, but the timing of the post-lunch questionnaire was reasonable (not more than 90 min after the restaurant opening time), the post-lunch time stamp was used and the pre-lunch stamp was discarded. 

Through the validated time stamps, objective stress values for the 180 min preceding the pre-lunch questionnaire and 180 min after the post-lunch questionnaire were identified and extracted from the activity bracelet data. Garmin Vivosmart 4 provides stress values every 3 min at its best, but movement by the user and low signal quality may hinder data collection and lead to missing values. To remove noise and impute missing values, the stress values were filtered with a Gaussian-weighted moving average filter with a window length of five samples, which corresponds to 12 min. The moving average filter slides a window over the samples and calculates an average over the samples in the window. The Gaussian-weighted filter gives less weight to samples which lie further from the center of the window. 

To study the evolution of stress values over time, the average and standard deviation of the filtered stress values for each time point were calculated for each study week based on all available data. For each participant, median values for stress were defined for periods of 180 min before lunch and for 1–90 min and 91–180 min after lunch. At least 30% of the potential data points per participant had to be available to calculate the median stress values. 

#### 2.5.2. Statistical Analyses

Repeated measures ANOVA with Bonferroni correction was used to study the differences in emotions and subjective stress between the study weeks for the subgroup of participants. The objective stress of the subgroup between the study weeks was analyzed with a paired samples *t*-test. For these analyses, the 180 min before lunch stress measures were averaged over all study weeks for all study participants and compared with both 1–90 min after lunch and 91–180 min after lunch stress measures. Correlations between emotions, subjective stress and objective stress were analyzed using Spearman’s rank correlation coefficient. A two proportions Z-test was used to analyze the differences in vegetarian and meat-based food choices of all customers between the study weeks and one-way ANOVA was used to analyze the amount of plate waste between the study weeks. Due to the real-life nature of the experiment, the criterion for statistical significance was set to a more liberal *p*-value of ≤ 0.1. The data were analyzed using IBM SPSS Statistics software (Version 24, IBM Corp, Chicago, IL, USA) and MATLAB R2020a (The MathWorks, Inc., Natick, MA, USA). 

## 3. Results

### 3.1. Emotions

Lunch experience in the ‘nature ambience’ evoked stronger positive emotions than that in the ‘fast food ambience’ (Table 1). However, no difference between ‘customary ambience’ and ‘nature ambience’ or ‘fast food ambience’ was detected. Eating lunch in ‘fast food ambience’ evoked stronger negative emotions than eating lunch in the ‘customary ambience’. No difference between ‘customary ambience’ and ‘nature ambience’ was detected in negative emotions.

### 3.2. Objective and Subjective Stress

#### 3.2.1. Objective Stress

**Figure 4:**
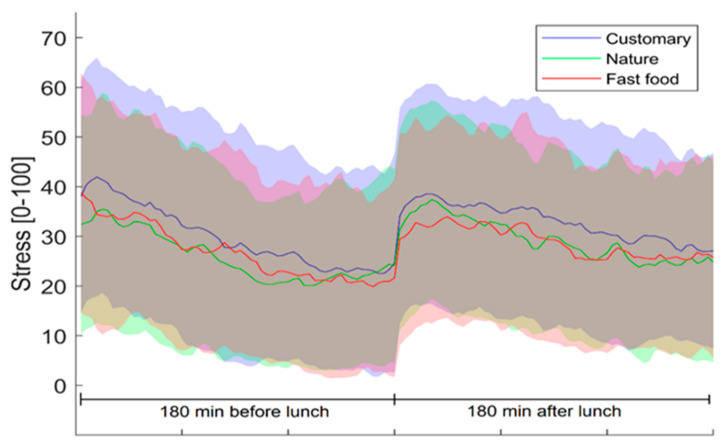
Evolution of objective stress over time in different ambiences on a scale in which 0–25 = resting state, 26–50 = low stress, 51–75 = medium stress, 76–100 = high stress. Solid lines show averages and shaded areas show standard deviations. Data from the subgroup of participants (*n* = 32).

Figure 4 shows how objective stress varied during the six hours of the working day around lunchtime (180 min before lunch and 180 min after lunch). Similar patterns were detected for all study weeks. In the morning hours, stress decreases until lunch. After lunch, there is a clear increase in measured stress. After the post-lunch peak, the stress slowly decreases towards the end of the working day. In general, stress levels in all weeks seem low.

**Figure 5:**
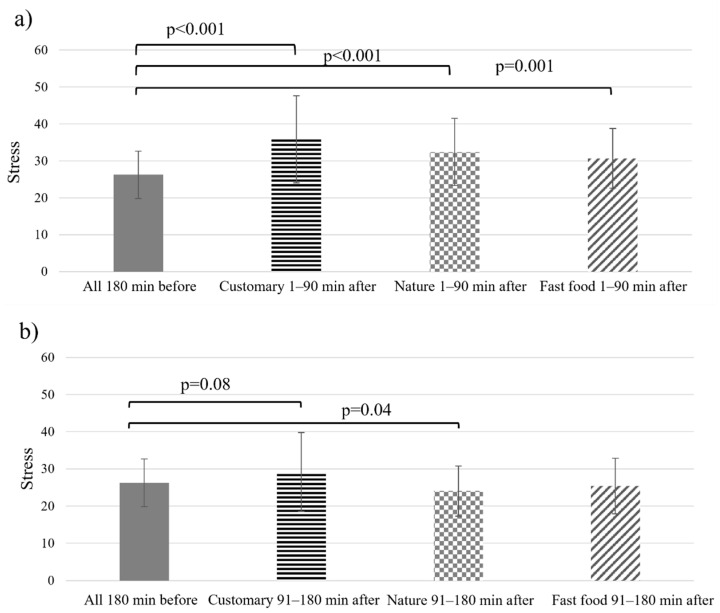
Averages of median objective stress 180 min before lunch (all weeks combined) and (**a**) 1–90 min after lunch and (**b**) 91–180 min after lunch. Scale: 0–25 = resting state, 26–50 = low stress, 51–75 = medium stress, 76–100 = high stress. Error bars are standard deviations of the medians. Data from the subgroup of participants (*n* = 32).

For the objective stress analyses, the before lunch stress levels were averaged over all study weeks for all participants to achieve a more valid baseline measure as the descriptive statistics before the lunch showed some unexpected variation between the study weeks (Figure 4). Figure 5a shows that stress was increased in each study week 1–90 min after lunch compared to 180 before lunch (all weeks combined), (‘customary ambience’, t(31) = −5.21, *p* = 0.000; ‘nature ambience’, t(31) = −4.62, *p* = 0.000; ‘fast food ambience’, t(31) = −3.82, *p* = 0.001). During the ‘nature ambience’ week, participants’ stress decreased 91–180 min after lunch compared to 180 min before lunch, t(31) = 2.15, *p* = 0.04. During the ‘customary ambience’ week, stress was still higher in 91–180 min period after the lunch in comparison to 180 min before lunch t(31) = −1.81, *p* = 0.08. (Figure 5b). 

#### 3.2.2. Subjective Stress

Subjective positive stress right before or after the lunch did not differ between the study weeks (Table 2). Subjective negative stress after the lunch was higher during the ‘fast food ambience’ week in comparison to the ‘customary ambience’ week.

#### 3.2.3. Correlations between Objective and Subjective Stress and Emotions

Table 3 presents correlations between objective stress, subjective stress, and emotions. In most cases, no statistically significant correlations were found between objective stress and subjective stress or emotions except for between objective stress and subjective negative stress before lunch during the ‘customary ambience’ week and after lunch during the ‘nature ambience’ week. Subjective stress correlated with emotions. Positive stress and positive emotions had positive correlations, negative stress and negative emotions had positive correlations, negative stress and positive emotions had negative correlations, and positive stress and negative emotions had negative correlations.

### 3.3. Food Choices

**Figure 6:**
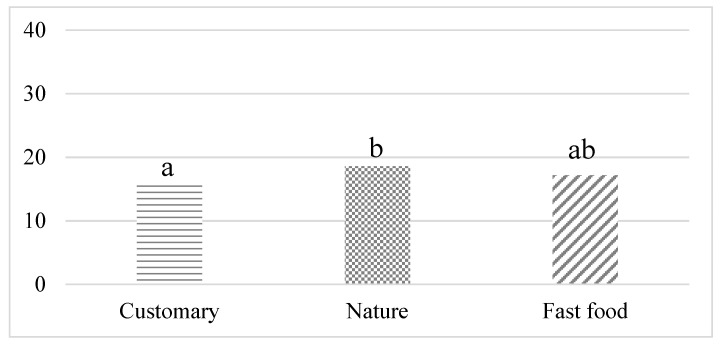
Relative share of vegetarian dishes of all lunch dish choices during the weeks with ‘customary ambience’, ‘nature ambience’ and ‘fast food ambience’. Data from all customers who purchased lunch during the study weeks (‘customary ambience’ week = 1610, ‘nature ambience week’ = 1714, ‘fast food ambience’ week = 1805). Different letters above the bars indicate statistically significant difference (*p* ≤ 0.1) between the study weeks.

Meat-based warm dish options were clearly the most popular dish options during all the study weeks (Figure 6). However, vegetarian lunch options were slightly more popular (18.6 %) during the ‘nature ambience’ week compared to the ‘customary ambience’ week (16.3 %), Z = −1.74, *p* = 0.08. No differences were detected between the ‘nature ambience’ week and the ‘fast food ambience’ week (17.2 %), Z = −1.08, *p* = 0.28 or between the ‘customary ambience’ and the ‘fast food ambience’ weeks, Z = 0.70, *p* = 0.48.

### 3.4. Plate Waste

**Figure 7:**
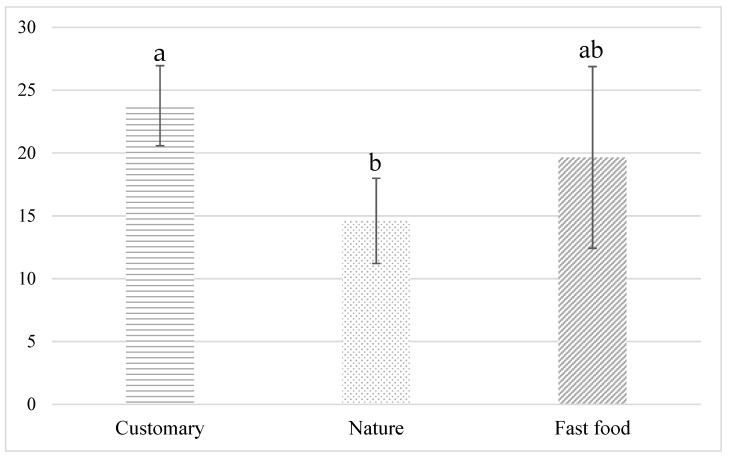
Average plate waste in grams per customer during the ‘customary ambience’, ‘nature ambience’ and ‘fast food ambience’ weeks. Data from each day of the study weeks (*n* = 15). Error bars are standard deviations of the five days of the study week. Different letters above the bars indicate statistically significant difference (*p* ≤ 0.1) between the study weeks.

On average, 23.8 ± 3.2 g, 14.6 ± 3.4 g, and 19.7 ± 7.2 g plate waste per customer was produced during the ‘customary ambience’, ‘nature ambience’ and ‘fast food ambience’ weeks, respectively, with some differences between the weeks [F(2,12) = 4.279, *p* = 0.04] (Figure 7). The Tukey post hoc test showed that the amount of plate waste was lower during the ‘nature ambience’ week in comparison to the ‘customary ambience’ week (*p* = 0.03). No statistically significant differences were detected between the ‘customary ambience’ and ‘fast food ambience’ weeks (*p* = 0.42) or the ‘nature ambience’ and ‘fast food ambience’ weeks (*p* = 0.28).

## 4. Discussion

The main expectation of this study was that different ambiences in a lunch restaurant can cause variation in customers’ positive or negative emotional responses and therefore influence stress recovery, food choices and sustainable behaviors. The results provided support to some of these assumptions. Partially in line with the expectations, the ‘nature ambience’ evoked stronger positive emotions than ‘fast food ambience’ and ‘fast food ambience’ evoked stronger negative emotions than the ‘customary ambience’. In terms of objective stress, a slight decrease in heart rate variability-based stress 91–180 min after lunch in ‘nature ambience’ was detected. No differences emerged in subjective stress between the study weeks except for a slight increase in negative after-lunch stress during the ‘fast food ambience’ week in comparison to the ‘customary ambience’ week. The correlations between positive and negative emotions and subjective stress were as expected, but surprisingly no correlations were found between emotions and objective stress or between objective and subjective stress. The data on food choices and plate waste showed that in ‘nature ambience’, the share of vegetarian dish choices was elevated and customers produced less plate waste in comparison to ‘customary ambience’.

In general, the results are to some extent in line with the earlier literature on restorative environments and their effect on emotional responses and stress recovery [1,16,17,27,28,29,30]. The ‘nature ambience’ developed for this study generated more positive emotional reactions than the ‘fast food ambience’. However, no differences were observed between ‘nature ambience’ and ‘customary ambience’. Although the effects of eating environment or context on consumers’ emotional responses have been demonstrated in many previous studies carried out in laboratory conditions [25,34,35], the differences between the ambiences in our field study were small despite the drastic differences observed in the pre-test. This might be because in a lunch restaurant setting, there are a multitude of other stimuli such as the lunch companion, restaurant layout, and rush that influence the situation simultaneously and might hide the experimental ambience effect. Possibly the effect could have been enhanced by bringing more elements such as plants or decorations in the restaurant. Both experimental ambiences could have brought a novelty aspect to the restaurant clientele as well, which could have hindered the desired effects on emotions. It is also possible that the customers were familiar with the customary ambience and changes to it in the first study week (‘nature ambience’) might not have been considered only as positive, which might explain why no differences in emotional responses were observed between the ‘customary ambience’ and ‘nature ambience’. Finally, emotional responses were measured only from a relatively small sample of study participants. With a larger sample, the results may have been more conclusive. 

Stress during working time was evaluated objectively with a wrist device that measures heart rate variability (HRV) and subjectively through questionnaires. ‘Nature ambience’ was expected to induce more positive emotional responses and therefore decrease stress. In each of the study weeks, objective stress slightly decreased during morning hours, increased clearly after lunch and gradually decreased after the post-lunch peak towards the end of the day. Measured stress increased in all study weeks when comparing the median stress of all weeks before lunch (180 min) to 1–90 min after lunch. This result can be explained by a meal-induced sympathetic activation, which is a natural consequence of eating. The activation was found to start soon (approx. in 15 min) after eating and last at least 90 min [36]. The properties of a meal (size, macronutrients) and individual characteristics (weight, age) influence the extent of meal-induced sympathetic activation as well. Coffee drinking [37], which is typical for Finnish people after lunch and moving back to office from the lunch restaurant most probably increased sympathetic activation as well. For these reasons, the stress values were observed not only straight after lunch but also 91–180 min after lunch when the meal-induced activation was expected to finish. In that time period (91–180 min after the lunch), objective stress in ‘nature ambience’ decreased compared to the combined pre-lunch stress from all weeks. This indicates that eating in ‘nature ambience’ might result in slightly decreased stress during the afternoon hours. However, the objective stress measures should be interpreted with caution. This is due to the fact that no variation was detected in subjective stress measurements between the study weeks. In addition, neither emotion measures nor subjective stress measures correlated with the objective stress measurements, which is illogical especially as the correlations between emotion measures and subjective stress measures were logical and according to expectations. These observations raise questions of how reliably HRV measurements with a wristband actually measure stress, how the data could be interpreted, and what type of conclusions can be drawn based on such data. 

The customers’ lunch choices and plate waste were recorded throughout the study weeks. The results show that the relative share of vegetarian dish choices was marginally higher and the amount of plate waste lower during the restorative ‘nature ambience’ week than the ‘customary ambience’ week. These are desirable changes since vegetable rich options are generally healthy and plate waste is unwanted from both ecological and financial perspectives. Previous research has shown that environmental cues are able to prime food choices. For example, a lunch buffet with green plants and odor of herbs nudged customers to take less food from the buffet line [38]. Even just an exposure to a nature poster increased healthy choices [8]. There is also evidence from laboratory experiments that exposure to nature videos leads to more sustainable behavior [19]. It can be assumed that the ’nature ambience’ in this study might have activated either a mental concept of a healthy diet or pro-environmental behavior leading to healthier and more sustainable choices. Whether these are separate or connected mechanisms in our study is debatable and requires further enquiries. For instance, the activation of a mental concept of a healthy diet might have led customers to choose smaller portions, a significant predictor of the amount of plate waste [39,40], which could have led to less plate waste, suggesting that the discovered results on food waste are mediated by the activated mental concept related to health. On the other hand, there is evidence that visual exposure to nature images or videos has a direct effect on sustainable behaviors as well [19]. However, the latter studies have not been carried out in a food consumption context and thus do not provide direct explanations on the matter. Finally, gender and food type [40,41] have also shown to influence the amount of plate waste. In the current study, the food types were controlled over the study weeks, but there is no data on the potential changes in gender composition of the restaurant clientele between the study weeks, which could have had an effect on the study results. To sum up, although the exact mechanisms behind the ambience effect on food choices and plate waste remain unclear, the study provides evidence that ambience modification in real life can have at least a moderate effect on healthier and more sustainable food behaviors.

### 4.1. Suggestions for Further Research

In the current study design, the expectation was that positive emotional responses to the eating environment would have beneficial effects on stress, food choices and plate waste. It must be noted that there is recent evidence that negative emotional appraisals might have a beneficial effect especially on sustainable food behaviors. For instance, a recent study found that a feeling of guilt leads to the reduction of household food waste [42]. Similar findings were introduced in a study related to meat consumption from a sustainability perspective [43]. These are interesting findings for further research aiming at understanding how eating environment modification affects food consumption behaviors.

This study focused on positive and negative emotions on a general level. However, it has been proposed that certain specific positive emotions such as joy and pride could lead to, for instance, environmentally sustainable behaviors [20]. Further research could consider priming specific emotional responses instead of the simpler valence of the emotions. Understanding which specific emotions are connected to healthy and environmentally sustainable behaviors would support the development of practical applications to facilitate consumers’ healthy and sustainable food consumption. 

The ambience modification was implemented through visual and auditory changes of the environment. Further research could test other measures of ambience modification such as olfactory ambience modification. Technological advancements in virtual reality tools could also offer interesting opportunities to develop innovative eating ambiences. 

### 4.2. Limitations of the Study

Five main limitations of the study deserve discussion. Firstly, the effect of the experimental ambiences on customers’ emotions was weaker than expected based on the literature and pre-test results. Stronger manipulation might have produced stronger effects on emotions, stress, food choices and amount of plate waste. In hindsight, it would have been useful to pre-test the chosen experimental images and sounds in the research restaurant environment instead of a more controlled paper and pen approach. Secondly, the curtains of the restaurant were kept closed during the study weeks to minimize the effect of changing weather and season on the study. Some of the customers might have been discontent with curtains being closed and it might have influenced how the participants perceived the lunch experience. Thirdly, there was a considerably large number of missing values (48% of the filtered data) in HRV based stress data, which needed to be balanced in the data pre-screening phase (see details in Section 2.5.1). Missing data was most probably due to participants’ movements and low signal quality. Similar type of wristband (Empatica) based HRV measurements have been shown to function well when the user is seated and in rest but not in dynamic conditions [44]. An HRV detection system with electrodes attached to the skin would have provided higher quality data, but in the context of a three-week study it would have been too burdensome for the participants. Consumer wearables have gained popularity during the past years also in the research field, but in practice their use is not optimal [45]. Fourthly, the sample subjected to emotion, subjective and objective stress measurements (*n* = 32) was relatively small, limiting the power of the study. Therefore, the results should be treated with caution and indicative at best. Despite the recruitment efforts, no more participants were attracted. The interest might have been low as participation in the study required long-term commitment by the participants and almost daily visits to the restaurant during the study weeks. Finally, there was imprecision in the timing when the participants filled in the pre and post lunch questionnaires. The participants were sent the questionnaires twice a day, but they had to remember to fill them in at the right time (right before and right after lunch). Some data were lost because of incredible time stamps. A more accurate way to collect the data would have been to send a notification to the participant at the moment they were entering or leaving the restaurant. 

Despite the limitations, the study has strengths. The main strength of this study is that it was conducted in a real-life environment, unlike many previous studies that have observed the impact of different stimuli on emotions, stress, food choices and sustainable behaviors in laboratory settings. In addition, this study approached the study topics with various methods including subjective measures, objective physiological measures (heart rate variability) as well as objective observations on food choices and amount of plate waste. In addition, the number of participants especially regarding food choices and plate waste (all the customers) was high, allowing strong conclusions.

## 5. Conclusions

A field experiment in a lunch restaurant was carried out to understand the effects of two experimental visual and auditory ambiences, ‘nature ambience’ intended to evoke positive emotions and ‘fast food ambience’ intended to evoke less positive emotions, on customers’ emotional states, recovery from stress, food choices and plate waste. The ambiences were designed to generate varying emotional responses with potential subsequent effects on the other measures and presented to customers through large projections on the walls and via loudspeakers. The results of the study indicate that ‘nature ambience’ in a lunch restaurant has at least a moderate positive effect on customers’ emotional states, vegetarian lunch choices and plate waste. The results also showed that under ‘nature ambience’, stress recovery 91–180 min after lunch was marginally elevated. However, the stress results should be interpreted with caution. To conclude, the study provides real-life evidence that restaurant visual and auditory ambience modification might lead to beneficial consequences among the clientele. 

## Figures and Tables

**Table 1:** Positive and negative emotions evoked by the lunch experience on a scale in which 1 = low intensity and 5 = high intensity. Data from the subgroup of participants (*n* = 32). The different letters next to the means indicate statistically significant difference (*p* ≤ 0.1) between the study weeks.

	Ambience				
	Customary	Nature	Fast Food	F (df)	*p* Value
Positive emotions, mean (SD)	3.2 ± 0.5 ^ab^	3.3 ± 0.7 ^b^	3.0 ± 0.7 ^a^	3.267 (2)	0.052
Negative emotions, mean (SD)	1.3 ± 0.4 ^a^	1.6 ± 0.7 ^ab^	1.6 ± 0.6 ^b^	4.376 (2)	0.024

**Table 2:** Subjective positive and negative stress just before and just after lunch during the study weeks measured on a scale in which 1 = low intensity and 5 = high intensity. Data from the subgroup of participants (*n* = 32). Different letters next to the means indicate statistically significant difference (*p* ≤ 0.1) between the study weeks.

	Ambience				
	Customary	Nature	Fast Food	F (df)	*p* Value
Subjective positive stress					
Just before lunch	2.8 ± 0.7	2.9 ± 0.7	2.8 ± 0.7	0.268 (2)	0.766
Just after lunch	2.8 ± 0.7	2.8 ± 0.7	2.7 ± 0.6	0.328 (2)	0.722
Subjective negative stress					
Just before lunch	2.1 ± 0.6	2.2 ± 0.6	2.2 ± 0.6	0.756 (2)	0.475
Just after lunch	1.9 ± 0.6 ^a^	2.1 ± 0.7 ^ab^	2.2 ± 0.6 ^b^	3.337 (2)	0.046

**Table 3:** Spearman’s rank correlations between objective stress, subjective stress and emotions evoked by the ambiences. Data from the subgroup of participants (*n* = 32). ** *p* ≤ 0.01, * *p* ≤ 0.1, ^ns^ non-significant correlation.

	Customary	Nature	Fast Food
	r	r	r
Before lunch			
Objective stress			
Subjective positive stress	−0.05 ^ns^	0.07 ^ns^	−0.08 ^ns^
Subjective negative stress	0.23 *	0.06 ^ns^	−0.03 ^ns^
After lunch			
Objective stress			
Subjective positive stress	−0.04 ^ns^	−0.15 ^ns^	−0.13 ^ns^
Subjective negative stress	0.13 ^ns^	0.24 *	−0.05 ^ns^
Positive emotions	−0.10 ^ns^	−0.21 ^ns^	−0.11 ^ns^
Negative emotions	0.10 ^ns^	0.21 ^ns^	0.15 ^ns^
Subjective positive stress			
Positive emotions	0.30 **	0.47 **	0.36 **
Negative emotions	−0.23 *	−0.19 *	−0.14 ^ns^
Subjective negative stress			
Positive emotions	−0.47 **	−0.42 **	−0.44 **
Negative emotions	0.37 **	0.44 **	0.38 **

## Data Availability

Data is available by request from the authors. The public availability of the data is restricted due to privacy and ethical reasons.
